# Transition to PCR diagnosis of cryptosporidiosis and giardiasis in the Norwegian healthcare system: could the increase in reported cases be due to higher sensitivity or a change in the testing algorithm?

**DOI:** 10.1007/s10096-022-04426-3

**Published:** 2022-03-04

**Authors:** Sophie M. Campbell, Frank O. Pettersen, Hanne Brekke, Kurt Hanevik, Lucy J. Robertson

**Affiliations:** 1 Parasitology, Department of Paraclinical Sciences, Faculty of Veterinary Medicine, Norwegian University of Life Sciences, NMBU Veterinærhøgskolen, Postboks 5003, 1432 Ås, Norway; 2 Present Address: Tallaght University Hospital, Dublin, Ireland; 3 Regional Advisory Unit of Imported and Tropical Diseases, Department of Infectious Diseases, Oslo University Hospital, Ullevål, Oslo, Norway; 4 National Reference Centre for Molecular Parasitology Diagnostics, Department of Microbiology, Oslo University Hospital, Ullevål, Oslo, Norway; 5 Norwegian National Advisory Unit on Tropical Infectious Diseases, Department of Medicine, Haukeland University Hospital, Bergen, Norway

**Keywords:** Cryptosporidiosis, Diagnostics, Giardiasis, Norway, Notifiable infection, Testing algorithm

## Abstract

Cryptosporidiosis has been a notifiable infection in Norway since 2012 and giardiasis since 1977. For both infections, there has been an increase in notified cases. We used a questionnaire to explore whether this may be associated with implementation of molecular diagnostic methods. We received responses from 14 of 16 laboratories, most of which had implemented molecular diagnostic methods for these parasites. Algorithms for testing had also been modified, and several laboratories now test more faecal samples than previously for both parasites. The increase in reported cases may reflect not only higher sensitivity of diagnostic methods, but also more sample testing.

**Supplementary Information:**

The online version contains supplementary material available at 10.1007/s10096-022-04426-3.

Cryptosporidiosis and giardiasis are gastrointestinal infections caused by the protozoan parasites *Cryptosporidium* spp. and *Giardia duodenalis* (syn. *Giardia lamblia*, syn. *Giardia intestinalis*), respectively. Both are associated with diarrhoea and abdominal discomfort, although subclinical infections and asymptomatic carriage also occur [1].

In Norway, all cases of cryptosporidiosis and giardiasis are notified to the Norwegian Institute of Public Health’s “Norwegian Surveillance System for Communicable Diseases” (MSIS; http://www.msis.no ). Cryptosporidiosis has been notifiable since 2012 and giardiasis since 1997.

Reported cases of cryptosporidiosis have increased since notification began, with the proportion of domestically acquired cases also rising, from 50% in 2012 to 73% in 2020 (Fig. 1). For giardiasis, reported case numbers vary annually, with an unusual peak in 2004 that was associated with a waterborne outbreak in Norway [2]. With the exception of 2004, the highest numbers of reported cases were in 2017, 2018, and 2019, and the lowest in the first 8 years (1977–1983) of cases being notified (Fig. 2).

**Fig. 1:**
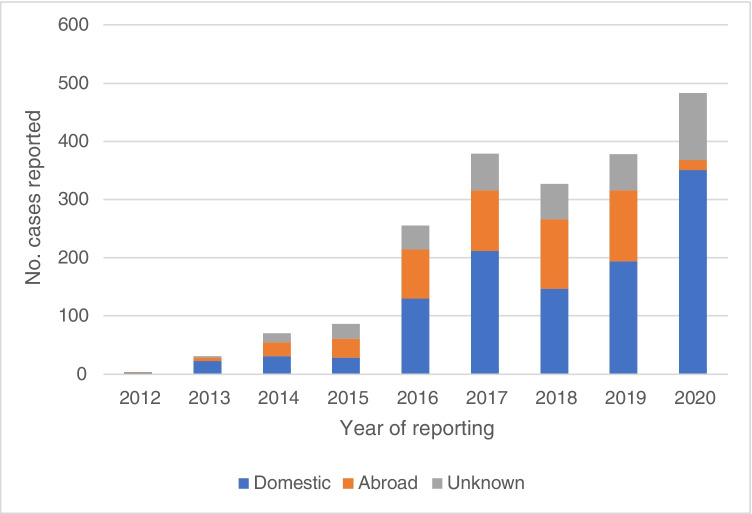
Number of cases of cryptosporidiosis reported to the Norwegian Surveillance System for Communicable Diseases (MSIS) from 2012 to 2020

**Fig. 2:**
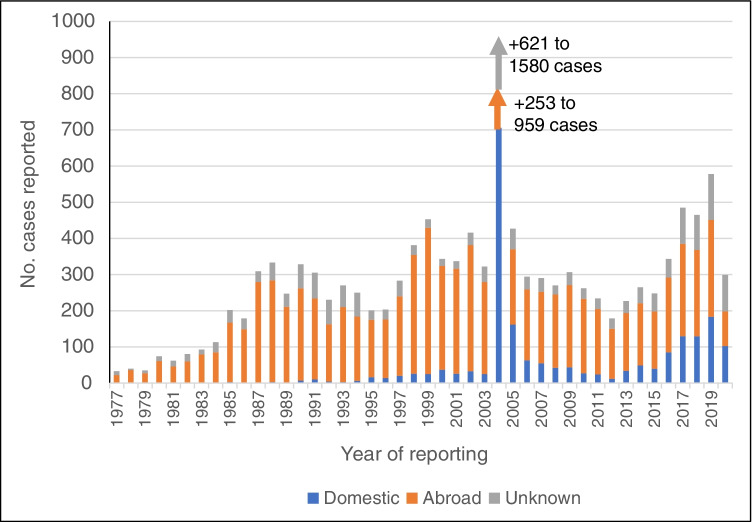
Number of cases of giardiasis reported to the Norwegian Surveillance System for Communicable Diseases (MSIS) from 1977 to 2020

Both diseases have traditionally been diagnosed by microscopy of faecal samples; a previous survey in Norway showed that molecular methods were not used for detection of *Cryptosporidium* or *Giardia* from 1998 to 2002 [3]. Of the 12 laboratories undertaking diagnostics for cryptosporidiosis at that time, most employed microscopy with modified Ziehl-Neelsen (mZN) staining, two also used phenol-auramine staining, one modified Kinyoun’s stain, three immunofluorescent antibody testing (IFAT), and four rapid immunochromatographic antigen tests [3]. Diagnosis of giardiasis was mainly based on light microscopy; two laboratories used IFAT, two rapid immunochromatographic antigen tests, and one an ELISA antigen kit [3].

In recent years, molecular methods have been implemented in routine diagnostics in many laboratories in Europe [4], including Norway. Advantages of these methods are speed, sensitivity, suitability for automation, and investigation for several pathogens in the same PCR setup (multiplex-PCR).

In Norway’s neighbouring country, Sweden, *Cryptosporidium* and *Giardia* infections are notifiable to the Swedish Public Health Agency [5–7]. Introduction of qPCR in Sweden during the last 5–10 years has been postulated to have resulted in a rise in reported protozoal infections due to more sensitive diagnostics and increased focus [7]. However, unlike in Norway, the number of reported giardiasis cases in Sweden has not increased in the last 5 years. Although falling from over 2300 cases in 1997 to around 1000 cases in 2011, the number of notified cases in Sweden has hovered between 1000 and 1500 from 2001 to 2019, with annual variations.

In Norway, the rise in cases of cryptosporidiosis and giardiasis reported to MSIS in recent years has also been attributed to introduction of more-sensitive qPCR tests [8]. However, there are other possible reasons for this. For example, it may reflect a real increase in number of infections, perhaps associated with increased travel activity and globalization, or may be due to more samples being analysed because test algorithms at medical microbiology laboratories have changed. For cryptosporidiosis, the number of samples analysed annually in the 2003 survey [3] was very low; 10 of 12 laboratories examined under 10 samples annually.

Here, we investigated how diagnosis of these parasitic infections in Norwegian microbiological laboratories has changed since the 2003 survey, regarding both techniques and indications for testing of samples. Using regional data, we also explored whether such changes may have influenced the increasing numbers of cases reported to MSIS.

Our study is based upon a questionnaire survey (see Supplementary Information 1) consisting of 14 questions. Information was requested on the regional origin of samples received, current and previous methods for examining samples for *Cryptosporidium* and/or *Giardia*, and whether selection criteria for analysing for these parasites had changed. A case scenario (Supplementary Information 1, Questions 12 and 13) was provided in which laboratories were asked whether a sample would be examined for these parasites, according to both previous and current testing algorithms.

Among the 16 laboratories that undertake diagnostics for these parasites in Norway, 14 laboratories completed the survey (see map of locations in Supplementary Information 2). The results reported below are from those laboratories that completed the survey.

The majority of laboratories answering the survey (79%) began analysing samples for both parasites more than 5 years ago. Most laboratories analysed samples from Eastern Norway (7/14; 50%) and Western Norway (4/14; 29%). At the time of our survey, 12 of the 14 laboratories had already implemented molecular methods for detecting these parasites, and most of these (8/12) had implemented these methods during the previous 5 years (2017–2020). Previous methods used included microscopy (10/12), IFAT (6/12), and/or immunochromatographic rapid antigen tests (2/12). Of the 12 laboratories now using molecular methods, three no longer performed the previous methods; the other nine primarily used molecular methods, but also offered conventional methods.

Of the 12 laboratories currently using molecular methods, none used their own in-house simplex PCR for individual parasites, but two used an in-house multiplex qPCR. Ten laboratories used a commercial qPCR kit for parasites or a pathogen-panel kit (see Table 1). Two laboratories offered different combinations of other non-molecular based methods; one laboratory used IFAT for *Cryptosporidium* and light microscopy and/or IFAT for *Giardia*, whereas the other laboratory used microscopy and rapid antigen tests for both parasites.

**Table 1:** Overview of commercially available diagnostic kits used by the laboratories

Kit	Number of laboratories using kit^1^
Multiplex qPCR kits for parasites	
Allplex™ GI-Parasite Assay from Seegene	4
FTD Stool Parasites from Fast Track	2
RIDA®GENE Parasitic Stool Panel from R-Biopharm	1
Viasure Multiplex from CerTest	1
Pathogen-panel kits	
EntericBio Dx Molecular GI Panel from SeroSep	1

^1^For one laboratory, the kit used was not named

Respondents were asked whether they would analyse for *Cryptosporidium* and/or *Giardia* in first-line testing, both now with current (molecular) methods and previously (with traditional methods), using a scenario in which the patient is an adult with persistent (> 14 days) diarrhoea, without underlying disease and no significant travel history. Whereas all laboratories that had implemented molecular methods would now test for both *Cryptosporidium* and *Giardia*, the two laboratories not using molecular methods would not test for either parasite.

For previous methods, seven laboratories answered that, in this scenario, they would not have tested for either parasite, three would have tested for both parasites, and two would have tested only for *Giardia*, but not *Cryptosporidium*. Again, the two laboratories currently not using molecular methods would also not have tested for these parasites previously.

Regarding algorithms for performing diagnostic tests for *Cryptosporidium* and *Giardia*, six laboratories test all samples for these parasites if the requesting doctor had asked for analysis for any intestinal pathogen. One of these laboratories also performs microscopy if the Ct value from qPCR is below 30, to help maintaining microscopy competence. Two laboratories responded that they examined for *Cryptosporidium* and *Giardia* if requested. Another laboratory tests for these parasites based on relevant travel history, prolonged diarrhoea, immunosuppression together with diarrhoea, or clinical suspicion. One laboratory tests for both *Cryptosporidium* and *Giardia*, except for when *Clostridioides difficile* toxin or norovirus analyses were requested for hospitalised patients.

Regarding former algorithms, nine laboratories responded that previously they only analysed faecal samples for *Cryptosporidium* and/or *Giardia* when explicitly requested. This was changed simultaneously with, or directly after, implementation of PCR-based diagnostics. In addition, seven laboratories changed the algorithm specifically because they had implemented PCR, as this provided a broader opportunity for investigation. Furthermore, one laboratory stated that one reason for implementing multiplex PCR was due to suspicion that cryptosporidiosis was considerably underdiagnosed.

It is difficult to compare regional reporting of cryptosporidiosis and giardiasis to MSIS and implementation of new diagnostic methods in that specific region, as some laboratories analyse samples from outside their own region. However, only one laboratory reported receiving samples from northern Norway, and only one analysed samples from Trøndelag region.

The laboratory analysing samples from northern Norway started using molecular diagnostic methods in 2020. From 1977 to 2019, the average number of annual cases of giardiasis reported to MSIS from this region was 19.2 (SD 15.4), but in 2020, 33 cases were reported. Equivalent data for *Cryptosporidium* was an average of 2.8 cases (SD 1.9; range 1–5) from 2014 to 2019, rising to 8 cases in 2020.

Results from Trøndelag were similar. Prior to introducing PCR-based diagnostic techniques in 2019, the annual average of reported giardiasis cases from this region was 32 (SD 23.9; range 4–199), increasing to an average of 57 (SD 14.2; range 45–69) after implementation. For cryptosporidiosis, an average of 6.5 cases (SD 6.4; range 2 to 11) were reported before implementation of molecular diagnostics, increasing to an average of 75 cases (SD 12.8; range 59–92) following implementation. Thus, the data suggest that changes in testing method and algorithm are associated with increased case reporting, particularly for cryptosporidiosis.

One main finding is that most medical microbiology laboratories in Norway that analyse samples for *Giardia* and *Cryptosporidium* have introduced molecular diagnostic methods for these parasites since the 2003 study [3]. This shift from traditional diagnostics to molecular methods has led to a much broader, more comprehensive testing for both parasites, especially in patients with no travel history. This is reflected in the MSIS data, where an increasing number of domestically acquired cases are reported, especially for cryptosporidiosis.

In 2012, when reporting of cryptosporidiosis to MSIS began, considerably more giardiasis cases were reported than cryptosporidiosis cases. In contrast, in 2020, the number of reported cryptosporidiosis cases (*n* = 483) was considerably higher than the number of giardiasis cases (*n* = 299). Significantly less travel activity in 2020 (due to COVID-19) probably contributed to a sharp decline in giardiasis, but this was not the case for cryptosporidiosis. Although the increase in reported cases of cryptosporidiosis may reflect implementation of PCR-based diagnostics, more patients with long-term immunosuppressive treatment may also have contributed. In addition, given that zoonotic *Cryptosporidium* occurs commonly among Norwegian livestock (e.g., [9–12]), greater countryside activity among the Norwegian population in 2020 may have contributed to more cases of domestic cryptosporidiosis.

Based on answers to the scenario question, together with comments regarding algorithm-based testing, both now and previously, it is clear that the number of faecal samples analysed regularly for *Cryptosporidium* and *Giardia* has increased significantly, often simultaneously with implementation of molecular methods. As 40% of laboratories participating in our study analyse all samples for intestinal pathogens for both *Cryptosporidium* and *Giardia*, a large increase in analyses for these parasites was expected. The role of microscopy in the parasite diagnostic laboratory is becoming increasingly restricted as molecular methods supplant them. Whereas microscopy is likely to remain the mainstay for these diagnostics in resource-constrained settings for some time to come [13], its role is likely to decrease in middle- and high-income countries for diagnosing cryptosporidiosis and giardiasis. However, for less common parasites, especially those not included in standard multiplex PCR panels, microscopy is likely to remain of importance, and also for analysis of non-clinical samples. It is possible that microscopy skills will become restricted to specialised teaching and research centres.

In other European countries, especially Sweden and the UK, the *Cryptosporidium* species in human infections is usually determined, often to subtype level, as this provides important epidemiological information [14]. Although Norway reported to European Food Safety Authority that the *Cryptosporidium* species is always determined from human cases [15], in practice, the infecting species is not usually investigated in sporadic cases. However, molecular characterisation may be conducted in outbreaks (e.g., [11, 12, 16]), as was also the case with *Giardia* isolates during the outbreak in Bergen in 2004 [2].

In conclusion, implementation of molecular methods for diagnosing cryptosporidiosis and giardiasis in Norway provides a better overview of the occurrence of both infections in Norway, including domestically acquired cryptosporidiosis. Although greater diagnostic sensitivity probably has a role, altered testing algorithms are probably as least as important. Consideration should be given to introducing a common strategy for testing samples for *Cryptosporidium* and *Giardia* in Norway. In addition, investigating positive findings with respect to species and genotype could provide further relevant information for understanding the epidemiology of infection and transmission routes.

## Supplementary Information

ESM 1 (DOCX 19 kb)

ESM 2 (JPG 120 kb)

## Data Availability

The original datasets (questionnaire responses) are not publicly available due to links to specific laboratories, but are available from the corresponding author on reasonable request.
