# Supplementary material for: Transition to PCR diagnosis of cryptosporidiosis and giardiasis in the Norwegian healthcare system: could the increase in reported cases be due to higher sensitivity or a change in the testing algorithm?
Source: Eur J Clin Microbiol Infect Dis. 2022 Mar 4;41(5):835–9. doi: 10.1007/s10096-022-04426-3 (PMC8893977; doi:10.1007/s10096-022-04426-3)
Supplement: Supplementary file 1 — (DOCX 19 kb) [file 10096_2022_4426_MOESM1_ESM.docx]

**Supplementary 1: Questionnaire (translated to English)**

**Transition to PCR diagnosis of cryptosporidiosis and giardiasis in the Norwegian healthcare system: could the increase in reported cases be due to higher sensitivity or a change in the testing algorithm?**

Sophie M. Campbell, Frank Olav Pettersen, Hanne Brekke, Kurt Hanevik, Lucy J. Robertson

1. **Background Information (free text)**
   1. Institute name:
   2. Your position in the lab:
   3. Email address
2. **From where do you receive samples (several answers possible)?**
   1. The whole of Norway
   2. Southern Norway
   3. Eastern Norway
   4. Western Norway
   5. Trøndelag (central Norway)
   6. Northern Norway
3. **Do you currently analyse samples for *Giardia* and *Cryptosporidium*?**
   1. *Giardia*, but not *Cryptosporidium*
   2. *Cryptosporidium*, but not *Giardia*
   3. Both *Cryptosporidium* and *Giardia*
   4. No
4. **When did you start analysing samples for *Giardia*?**
   1. In the last 12 months
   2. In the last two years
   3. In the last 5 years
   4. Longer ago
5. **When did you start analysing samples for *Cryptosporidium*?**
   1. In the last 12 months
   2. In the last two years
   3. In the last 5 years
   4. Longer ago
6. **Do you use molecular methods (e.g., PCR) for analysing samples for these parasites?**
   1. Yes for *Cryptosporidium*
   2. Yes for *Giardia*
   3. Yes for both
   4. No
7. **Which types of molecular methods (several answers possible)?** (this question only for those who answered «Yes» to question 6)
   1. Internal simplex qPCR for *Cryptosporidium*
   2. Internal simplex qPCR for *Giardia*
   3. Internal multiplex qPCR that includes *Giardia* and *Cryptosporidium*
   4. Commercial qPCR kit for *Cryptosporidium* (e.g., Viasure, ViPrime etc.)
   5. Commercial qPCR kit for *Giardia* (e.g., Viasure, ViPrime etc.)
   6. Pathogen-panel kit (e.g., FilmArray, xTag, etc.)
   7. Specify which kits are used. If other molecular methods (e.g., NASBA, LAMP etc.) are used, please describe per parasite.
8. **When did you implement the current method (MM / YY)?** (this question only for those who were directed to question 7)
   1. For *Cryptosporidium*
   2. For *Giardia*
9. **Which other methods did you use previously (several answers possible)?** (this question only for those who were directed to question 7)
   1. Microscopy (light, with or without staining e.g. iodine) for *Giardia*
   2. Microscopy (light, with or without staining e.g. modified Ziehl Neelsen) for *Cryptosporidium*
   3. Immunofluorescent antibody test for *Giardia*
   4. Immunofluorescent antibody test for *Cryptosporidium*
   5. Rapid antigen test for *Giardia*
   6. Rapid antigen test for *Cryptosporidium*
   7. We have only ever used our current method
   8. Rapid antigen test for Giardia
   9. Other methods (e.g, previous molecular methods etc. (please describe))
10. **Do you still offer these previous methods?** (this question only for those who reported using other methods previously in question 9)
    1. No
    2. Yes, but we primarily use our molecular methods
    3. Yes, we use these methods primarily, and molecular methods for confirmation
11. **Which methods do you use?** (this question only for those who answered «No» to question 6)
    1. Microscopy (light, with or without staining e.g. iodine) for *Giardia*
    2. Microscopy (light, with or without staining e.g. modified Ziehl Neelsen) for *Cryptosporidium*
    3. Immunofluorescent antibody test for *Giardia*
    4. Immunofluorescent antibody test for *Cryptosporidium*
    5. Rapid antigen test for *Giardia*
    6. Rapid antigen test for *Cryptosporidium*
    7. We have only ever used our current method
    8. Rapid antigen test for Giardia
    9. Other methods (please describe)
12. **If you were to receive a sample today from an adult patient with persistent diarrhoea (over 14 days) without underlying disease or significant travel history, would you test for *Giardia* and / or *Cryptosporidium* as part of the first-line test?**
    1. Yes, for both *Cryptosporidium* and *Giardia*
    2. Yes – only for *Cryptosporidium*
    3. Yes – only for *Giardia*
    4. No, for neither *Cryptosporidium* nor *Giardia*
13. **If you previously used another method, and received a sample as described in the previous question (an adult patient with persistent diarrhoea (over 14 days) without underlying diseases or significant travel history) you would have tested for *Giardia* and / or *Cryptosporidium*** **as part of the first-line test**
    1. Yes, for both *Cryptosporidium* and *Giardia*
    2. Yes – only for *Cryptosporidium*
    3. Yes – only for *Giardia*
    4. No, for neither *Cryptosporidium* nor *Giardia*
    5. We have always used the same method / Not applicable
14. **If you use an algorithm for testing *Giardia* and *Cryptosporidium*, please describe it (free text) If this has changed after the implementation of molecular methods, please describe this change (free text).**
    1. Current algorithm
    2. Previous algorithm (from which the current algorithm was changed) (
    3. Year for changing the algorithm for *Giardia*
    4. Year of change in the algorithm for *Cryptosporidium*
    5. Why was the algorithm changed for *Giardia* (new method, new information available, etc.)?
    6. Why was the algorithm changed for *Cryptosporidium* (new method, new information available, etc.)?
    7. Other comments (free text)
