# Supplementary figures and images for: Transition to PCR diagnosis of cryptosporidiosis and giardiasis in the Norwegian healthcare system: could the increase in reported cases be due to higher sensitivity or a change in the testing algorithm?
Source: Eur J Clin Microbiol Infect Dis. 2022 Mar 4;41(5):835–9. doi: 10.1007/s10096-022-04426-3 (PMC8893977; doi:10.1007/s10096-022-04426-3)

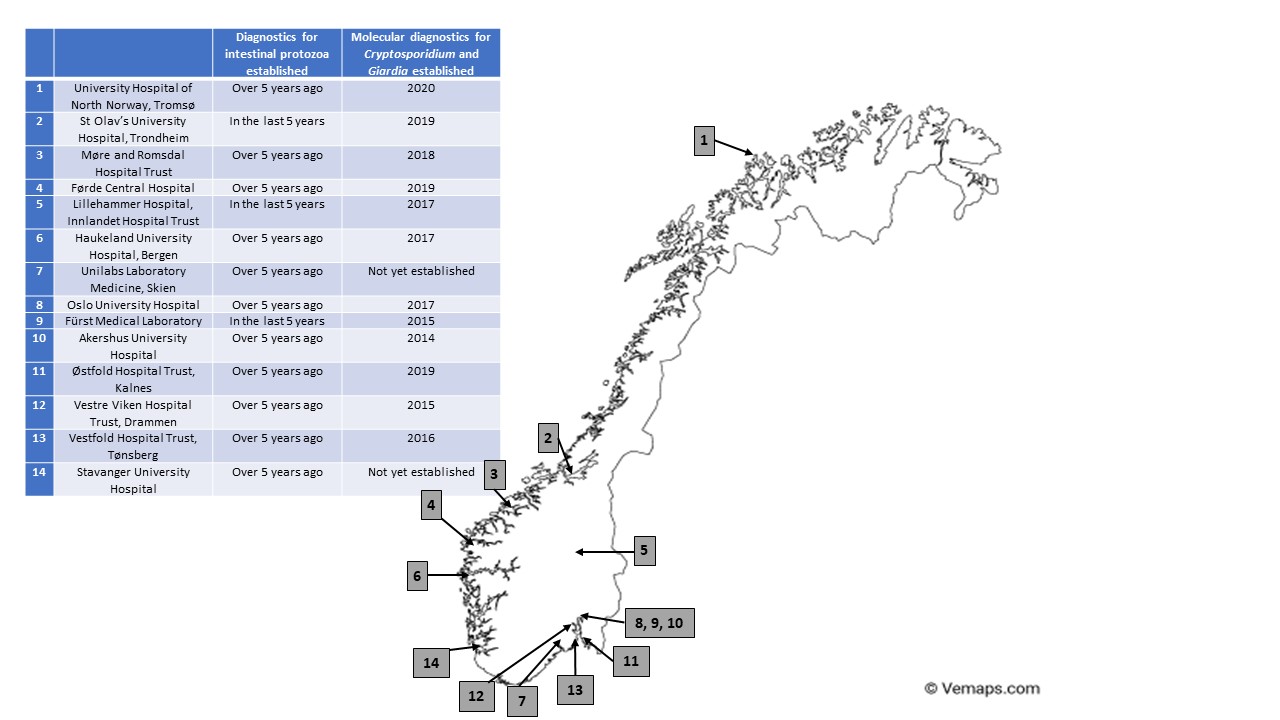

Supplement: Supplementary file 2 — (JPG 120 kb) [file 10096_2022_4426_MOESM2_ESM.jpg]
